# Detailed Analysis and Road Map Proposal for Care Transition Records and Their Transmission Process: Mixed Methods Study

**DOI:** 10.2196/60810

**Published:** 2025-02-21

**Authors:** Elisabeth Veronica Mess, Matthias Regner, Sabahudin Balic, Lukas Kleybolte, Lisa Daufratshofer, Andreas Mahler, Sabrina Tilmes, Viktor Werlitz, Claudia Reuter, Alexandra Teynor

**Affiliations:** 1 Institute for agile Software Development Technical University of Applied Sciences Augsburg Augsburg Germany; 2 Digitization Staff Unit University Hospital Augsburg Augsburg Germany

**Keywords:** care transition record, transmission management, observations, process modeling, telematics infrastructure, TI, Fast Healthcare Interoperability Resources, FHIR, Health Level 7, HL7, medical information object, MIO, care information object care transition record, CIO-CTR, Pflegerisches Informationsobjekt-Überleitungsbogen, PIO-ULB, artificial intelligence, AI

## Abstract

**Background:**

The digitalization of health care in Germany holds great potential to improve patient care, resource management, and efficiency. However, strict data protection regulations, fragmented infrastructures, and resistance to change hinder progress. These challenges leave care institutions reliant on outdated paper-based workflows, particularly for patient data transmission, despite the pressing need for efficient tools to support health care professionals amid a nursing shortage and rising demand for care.

**Objective:**

This paper aims to analyze Germany’s care transition record (CTR) and CTR transmission process as part of transition management and suggests improvements toward a seamless digital solution.

**Methods:**

To understand the current challenges of manual CTR transfers, we used a mixed methods approach, which included a web-based questionnaire with nursing professionals, field observations, business process model and notation modeling, semantic and frequency analysis of CTR entries, and user story mapping.

**Results:**

A web-based questionnaire involving German nursing professionals (N=59) revealed considerable delays in patient care due to manual, patient-transferred CTRs. Of the 33 usable responses (n=33), 70% (n=23) of the respondents advocating for digital transmission to improve efficiency. Observations (N=11) in care facilities (n=5, 45%) and a hospital (n=6, 55%) confirmed the high administrative burden, averaging 34.67 (SD 10.78) minutes per CTR within a hospital and 44.6 (SD 20.5) minutes in care facilities. A semantic analysis of various CTRs (N=4) highlighted their differences and complexity, stressing the need for standardization. Analyzing a new CTR standard (care information object CTR) and manually mapping an existing CTR to it showed that the procedure was ambiguous, and some associations remained unclear. A frequency analysis of CTR entities revealed which were most used. In addition, discussions with care staff pointed out candidates for the most relevant entities. On the basis of the key findings, a stepwise transition approach toward a road map proposal for a standardized, secure transfer of CTRs was conceptualized. This road map in the form of a user story map, encompassing a “CTR transformer” (mapping of traditional CTRs to a new standard) and “care information object CTR viewer/editor” (in short, CIO-CTR viewer and editor; a new standard for viewing, editing, and exporting), shows a possibility to bridge the transition time until all institutions fully support the new standard.

**Conclusions:**

A future solution should simplify the overall CTR transmission process by minimizing manual transfers into in-house systems, standardizing the CTR, and providing a secure digital transfer. This could positively impact the overall care process and patient experience. With our solutions, we attempt to support care staff in their daily activities and processes until nationwide state regulations are implemented successfully, though the timeline for this remains uncertain.

## Introduction

### Digitalization in Health Care in Germany

Digitalization has emerged as a transformative force across various sectors, fundamentally altering organizational operations and service delivery. Health care is one sector benefiting significantly from digitalization as it can support patient care, resource management, and overall efficiency [1,2].

The growing shortage of qualified nursing personnel and the rising number of people needing care signify the need for more efficient, high-quality processes and tools to support health care professionals. Digital solutions offer a pathway to address these challenges by automating administrative tasks, improving communication between health care providers, and freeing up valuable time for direct patient care [3-5]. In Europe, policy makers, researchers, and health care practitioners are working to enhance health care infrastructure and promote interoperability to foster more efficient and coordinated care [6]. However, in Germany, the digital transformation of health care remains slow and faces significant obstacles [3,7,8].

Stringent data protection regulations for the processing of personal data (eg, the European Union’s General Data Protection Regulation [GDPR] and Germany’s Patient Data Protection Act, derived from the GDPR [9]) and fragmented technical infrastructures combined with the resistance to change make it difficult to integrate new tools or adjust existing processes [7,10]. In addition, the lack of a unified digital strategy further hinders the seamless implementation of digital health solutions [7,10].

Ultimately, the complexity of implementing digital solutions in the German health care system stems from balancing innovation with regulatory compliance, data security, and protecting patient privacy.

### Care Transmission Process in Germany

A critical challenge within health care digitalization is ensuring the seamless transition of patient information between health care institutions. Paper-based workflows, still prevalent in many facilities, often cause delays and data loss during the transfer process due to the lack of standardized formats and the inability to share data in time.

Our research focuses on streamlining parts of the care transition record (CTR) transmission process to address this issue. The project’s goal is to improve the transfer of patient data across care institutions, which currently suffer from time-consuming manual data entry, format inconsistencies, and delays in the arrival of crucial patient information.

### State of the Art

#### Health Care Data Exchange

A security-conformant approach for digital transfer is the use of a dedicated health data (transfer) network. In Europe, such a service must be conformant to the GDPR, that is, legal compliance (ensuring data privacy and security), patient data control (data consent management for patients), data security (only access by authorized users, protection against breaches), and interoperability (fostering data exchange between different health care providers across various platforms) [9].

The telematics infrastructure (TI) is Germany’s digital health data network designed to connect all health care providers, enabling the exchange of medical data across institutions [11]. It integrates various applications to streamline communication between health care entities such as physicians, hospitals, and pharmacies.

A specific way to exchange health data within a health data network is via an electronic health record. An electronic health record represents the digital version of a patient’s medical history maintained over time by health care providers. It includes key clinical data relevant to patient care, such as medical history, diagnoses, medications, treatment plans, immunization dates, allergies, radiology images, and laboratory results. It is possible to share the patient data with other health care stakeholders, including the patient [12,13].

Several countries have made significant progress in this area, for example, the electronic patient dossier from Switzerland [14,15], electronic health record (Elektronische Gesundheitsakte) from Austria [16], MyKanta from Finland [17,18], and Mon espace santé from France [19]. They offer structured consent management for patients, meet the high security standards of the European Union, and foster interoperability by using the standard Fast Healthcare Interoperability Resources Health Level 7 (HL7), for exchanging electronic health care data.

The implementation of Germany’s electronic patient record (ePA) [20] is progressing; however, it faces challenges. Many health care providers are not yet integrated, and patients must manually upload data. Technical and privacy issues, including interoperability concerns and strict data protection laws, continue to hinder broader adoption and use [21,22].

Another component within the TI is Kommunikation im Medizinwesen (KIM). It is a communication service with which health data can be exchanged directly by care providers, such as via email [23]. Nationwide implementation of the TI has been slow due to interoperability challenges. Adoption has lagged, primarily due to concerns over complexity, costs, and workflow disruptions. Health care professionals are hesitant to fully transition to digital tools because of these technical difficulties and the perceived burden of TI integration. There are 2 model projects in Germany [24,25] piloting and evaluating the TI and including components (eg, KIM and ePA). Unfortunately, no detailed evaluation reports have been published yet.

#### Standardization of Health Care Data

Standardization is a significant aspect that can improve the transfer of patient data in terms of reducing potential manual data entry and format inconsistencies. Standardizing CTRs is a potential possibility for improving the CTR transmission process. In Germany, 2 subsequent projects have focused on this issue: the first project is the ePflegebericht.

The ePflegebericht Project (electronic nursing report) began in 2002 when the Network for Continuity of Care in the Osnabrück Region [26] developed the concept for an electronic nursing report [27]. Insights from testing this software and its transition forms were gathered in a project under the patronage of the German Nursing Council starting in 2006. These insights were generalized beyond local use, placed in an international context [28], and aligned regionally and nationally [29]. The result was then submitted for approval as an HL7 standard [30].

The ePflegebericht served as a data exchange format for sharing information between care facilities and hospitals. It is based on the HL7 Clinical Document Architecture standard described in the study by Flemming et al [31]. The study validated the HL7 Clinical Document Architecture–based ePflegebericht and confirmed that it could cover all relevant nursing data compared with 114 paper-based nursing summaries used by 806 health care facilities in Germany. The ePflegebericht provided a comprehensive structure for transferring nursing information, demonstrating its applicability during care transitions. It improved the transmission of nursing data compared to paper-based methods, adding details such as social and homecare information, leading to more holistic documentation. Technically, advancements such as reusable templates were also introduced. These updates led to the relaunch of the ePflegebericht, with slight modifications, and in 2019, it was again up for approval.

The introduction of the ePflegebericht marks a significant advancement in the standardization of CTRs in Germany. A nationwide initiative aiming to develop a standard format for a variety of health-related documents (ie, medical information objects [32]) used the ePflegebericht as a foundational model for their CTR format: Pflegerisches Informationsobjekt-Überleitungsbogen (PIO-ULB). In this paper, the authors refer to the PIO ULB as care information object (CIO) CTR. This initiative was commissioned by the German government and overseen by the Gesetzliche Krankenversicherung Spitzenverband (central representative body of the statutory health and nursing care funds in Germany), and the mio42 GmbH (organization that develops medical information objects on behalf of the National Association of Statutory Health Insurance Physicians [Kassenärztliche Bundesvereinigung]). Furthermore, it involved the collaboration of the Deutscher Berufsverband für Pflegeberufe eingetragender Verein (DBfK; German Professional Association for Nursing Professions eingetragender Verein), and the Deutscher Pflegerat eingetragender Verein (German Nursing Council eV) [32].

CIO-CTR uses HL7 Fast Healthcare Interoperability Resources datasets, as described in the publication by mio42 GmbH [33], and was completed by the end of 2022. However, there is still uncertainty regarding the swift implementation of this new standard, primarily due to the financial and human resource challenges faced by health care software manufacturers who must adapt their existing products to comply with the specification, which spans approximately 2000 pages (XML code), as shown in mio42 GmbH [34]. The CIO-CTR will be effective at the beginning of 2025 [34] but without legal obligation for software manufacturers to implement it.

### Objectives

This paper aims to analyze and address the challenges of the CTR transmission process in Germany. On the basis of a review of the current situation and possible approaches, a road map toward a fully digital, seamless solution is to be proposed. The overall goal is to improve the transfer of patient care data across care institutions.

## Methods

Several methods were used to assess the satisfaction of nursing staff in the context of patient data transfer in care facilities in Germany. These include the creation of a web-based questionnaire, conducting field observations and contextual inquiries, business process model and notation (BPMN) modeling, semantic and frequency analysis of existing CTRs, and user story mapping. The findings are presented in this paper.

### Web-Based Questionnaire

A web-based survey was conducted to identify challenges and preferences related to the CTR transmission process. The survey targeted nurses, nursing assistants, and trainees working in ambulatory, acute inpatient (eg, hospitals), or long-term care settings familiar with the CTR process. Participation was solicited through various channels, including the Bavarian State Ministry of Health and Prevention and the professional networks of project members. Due to a low initial response rate, the survey period was extended, and multiple reminders were issued. Using LimeSurvey, the survey ran from February 11, 2022, to April 30, 2022.

The questionnaire, developed iteratively by the project team (developers; care managers; and ethical, legal, and social issue experts), was based on literature and included custom questions and items from the validated Copenhagen Psychosocial Questionnaire tool [35]. Copenhagen Psychosocial Questionnaire items covered 12 domains, such as sociodemographic information (eg, gender, age, and work setting). Additional items focused on the experience with CTR creation and transmission, error rates, and attitudes toward digitalization. The 24-item questionnaire primarily used 4-point Likert scales, supplemented by nominal, metric scale, and open-ended questions. Respondents could opt out at any time, and all data were anonymized. A pretest with 7 participants from 2 independent institutions (implementation and nursing sciences) identified several structural and technical issues, which were addressed in a second pretest round. The same individuals tested the final version and did not reveal any issues.

Data analysis was conducted using SPSS (version 28.0.0.0; IBM Corp). Responses to open-ended questions were categorized using Microsoft Excel, and the data were checked for erroneous entries before being analyzed, focusing on descriptive statistics.

### Field Observations and Contextual Inquiries

#### Overview

Field observations and contextual inquiries were conducted in a hospital and inpatient care facilities to understand the CTR transmission process thoroughly. These methods focused on the activities of care staff in their natural work environments, providing foundational insights for process modeling and research. The CTR transmission process in this study refers to all activities involved in creating a CTR at the sending facility and integrating it into the in-house system at the receiving facility, including the use of computer equipment, work tools, and telephone calls, while accounting for potential confounding factors. The observations aimed to clarify whether staff entered all data from the CTR at once or alternated between tasks.

#### Field Observation

Field observation, a qualitative research method, involves systematically observing participants in their natural settings to collect rich, contextual data on behaviors, interactions, and the surrounding environment [36]. An observation protocol was established to ensure consistency across sites and sessions, focusing on key areas such as activities performed, use of aids (eg, software and hardware), how information was handled and transferred, and any special features or abnormalities. Unobtrusive observation techniques were used to minimize observer effect, and detailed field notes were recorded, capturing both activities and nonverbal cues.

#### Contextual Inquiry

Contextual inquiry, a user-centered design method, was used to observe participants in their natural work environments while engaging in informal conversation to ask questions or clarify processes. This approach provided a deep understanding of the context in which tasks were performed and the challenges faced by users [37,38]. These inquiries, which were conducted primarily in participants’ offices, allowed researchers to ask questions during task performance, facilitating an exploration of thought processes and decision-making, particularly with complex systems.

#### Execution

The observations and inquiries were conducted by 2 researchers, one with a medical background and the other specializing in user-centered design, ensuring comprehensive documentation and minimizing potential biases. The field observations and the contextual inquiries followed the same protocol. Thematic analysis [39] was applied to the data, with the researchers collaboratively reviewing and coding field notes to identify relevant patterns that informed the process modeling. In less formalized care facility environments, contextual inquiries were preferred, with researchers assuming an apprentice role to ask clarifying questions without disrupting workflows.

The observations were restricted to on-site care staff and did not include patients or external personnel (eg, patient transport). Observations occurred between 2020 and 2022, with no specific temporal or spatial restrictions within the facilities. Each observation was planned for 1 hour each.

### Ethical Considerations

All studies adhered to ethical guidelines, and informed consent was obtained from participants. All data were anonymized. No incentives were offered. Ethics approval for the study was granted by the joint ethics committee of the Universities of Bavaria (GEHBa-202107-V-028).

### BPMN Modeling of CTR Transmission Process

BPMN is an established and widely used graphical representation for modeling business processes. It is a standard developed by the Object Management Group (OMG) and has been adopted as an International Organization for Standardization (ISO) standard.

In BPMN, a process is represented as a sequence of activities or events, ordered in a flow that can be split or merged using gateways, directing the flow into one or multiple paths. Due to its simplicity, business process managers have widely used this standard in many application domains. Despite not being explicitly designed for clinical processes, BPMN has proven its value in the health care domain, allowing an easy-to-understand representation of clinical processes [40,41].

### Semantic Analysis of CTRs

Semantic analysis is a good approach to extract and interpret the meaning of terms and sentences in detail. In the discipline of computer science, it is a fundamental component of natural language processing [42,43].

For semantic analysis, CTRs (empty and filled with fictive patient data) from cooperation facilities (n=4) were analyzed and compared in detail to better understand their structure, similarities, and differences. For clarification of any questions (eg, exact meaning, relevance, or scope of a specific category or word and overall comprehension), 1 meeting per facility with care staff was held. Given the semistructured to unstructured nature of the CTRs, it was critical to determine which data elements hold the same or different information compared to another facility. The meetings (n=4) lasted approximately 60 minutes.

Afterward, the CTRs were mapped to the new CIO-CTR standard. For this, the CTR entries were subdivided into entities and values and afterward mapped with pen and marker to the new standard format CIO-CTR.

### Frequency Analysis of CTR Entities

Frequency analysis [44] is a method used to determine how often specific elements occur within a dataset, both in absolute terms and as a proportion of the total data. In this study, frequency analysis was applied to assess the occurrence of individual CTR entities to determine which pieces of information are most included. This helped inform the design of the proposed digital solution, ensuring that it prioritizes the most frequent CTR entries.

### User Story Mapping

User story mapping [45] is a user-centric bottom-up technique used to outline a product or product feature. The output, known as a story map, provides a global view of the product, detailing the steps a user takes to achieve a specific outcome. This method helps prioritize tasks, identify dependencies, and adapt to changes.

Story maps are organized along 2 dimensions: the backbone (horizontal axis), which represents the user’s activities step by step, and the release dimension (vertical axis), which defines the scope of the product and its various stages of development. A commonly used format for user stories is the role-feature-reason format: “As a <user>, I want to <feature> so that <value>” [45]. While a story backlog lists user stories in isolation, user story mapping provides a structured, global view of the entire application, fostering a common understanding between developers and stakeholders. This method also encourages communication, helping to eliminate misunderstandings early in the development cycle.

In the story mapping workshop, results from previous requirement analysis—including user feedback, product vision, and initial process modeling—are used to create actionable user stories. The key objectives of the workshop included understanding the user’s perspective, identifying potential gaps, prioritizing, and release planning.

In total, 2 workshops were conducted, involving a total of 7 participants. These participants were part of the core research project team, bringing diverse expertise from various disciplines: health care (n=2, 29%), computer science (n=3, 43%), design (n=1, 14%), and IT security (n=1, 14%). All 7 (100%) participants attended both workshops, ensuring continuity and consistency in the discussions and decisions.

## Results

### Web-Based Questionnaire

A total of 59 participants participated in the web-based survey to determine the experiences and needs of nursing professionals regarding care transition reports, of which 35 (59%) met the inclusion criteria. Of the 35 participants, 2 (6%) did not finish the survey, resulting in 33 usable datasets. In Table 1, specific sociodemographic information about the participants is provided. An overview of the systems or software used is also provided in Table 2.

**Table 1:** Sociodemographic information of participants (n=33).

Characteristics		Participants, n (%)
**Gender**		
	Women	22 (67)
	Men	10 (30)
	Nonbinary	1 (3)
**Age group** **(y)**		
	18-24	2 (6)
	25-34	13 (40)
	35-44	7 (21)
	45-54	8 (24)
	>55	3 (9)
**Care setting**		
	Short-term care (outpatient)	2 (6)
	Acute inpatient care (hospital)	28 (85)
	Long-term care (care facility)	3 (9)
**Federal state (within Germany)**		
	Bavaria	32 (97)
	Berlin	1 (3)

**Table 2:** Information about the system or software used.

Information			Participants, n (%)
**System or software used for the creation of CTRs^a^**			
	I use software	20 (61)	
	I do not know	4 (12)	
	I use a paper form	5 (15)	
	I use a paper form and software	3 (9)	
	Not specified	1 (3)	
**Specific software used**			
	ORBIS (by Dedalus)	16 (49)	
	C&S	1 (3)	
	SAP	1 (3)	
	Sic Pflegeassistent (by CGM SYSTEMA SIC)	1 (3)	
	SnapAmbulant (by euregon)	1 (3)	
	Sorian	1 (3)	
	Not specified	12 (36)	

^a^CTR: care transition record.

The high percentage of female participants (22/33, 67%) reflects the well-established predominance of women in nursing. The concentration of participants in the 25 to 34 age group suggests that the web-based survey may have been more appealing or accessible to younger adults. In addition, during the COVID-19 pandemic, care professional faced more stress and work, which might have led to a discouragement of answering a questionnaire that does not benefit their daily work.

The overwhelming representation of acute inpatient care (28/33, 85%) indicates a strong representation of hospitals in the questionnaire.

Of all the federal states in Germany, approximately all participants were from Bavaria (32/33, 97%) and only very few from Berlin (1/33, 3%). The overall overwhelming representation from Bavaria is probably due to the location of the research team, indicating that the recruiting efforts were particularly successful in this region despite numerous efforts to reach other care facilities and hospitals.

The results of the system or software used (Table 2) show that most (20/33, 61%) participants used software to create CTRs. Only 15% (5/33) of the participants used the paper form. Most (16/33, 49%) of the participants used the software ORBIS, reflecting the very high percentage of participants from hospitals, as ORBIS is a hospital information system. Sorian (1/33, 3%) is also a hospital information system. The other software listed (C&S, SAP, Sic Pflegeassistent, and Snap Ambulant) are documentation software used in the care facilities setting, which underlines the variety of software used.

Additional findings from the web-based questionnaire revealed that the CTRs were mainly transferred via the patient (27/33, 82%). This means that in these cases, the nurse gave the CTR to the patient as a printout, and the patient or the relatives were responsible for ensuring that it reached the next care facility.

As a result, the nursing staff at the receiving facility has limited time to fully prepare for the patient in advance. Preparations and admission begin once the patient arrives at the facility, which can lead to waiting times. This is consistent with the results from the field observations that were conducted. The remaining 18% (5/33) transferred the CTR via fax, patient file, or telephone.

This gives the nursing staff more time to prepare for the patient, for example, preparing for isolation, special therapy treatment, or similar. According to the survey, the manual transfer of the CTR into the in-house system takes an average of 45 minutes, and 61% (20/33) of care staff perceived the transfer process as time-consuming. Manual transfer means that the care professional copies the information from the printout using their hand (typing on the keyboard) and transfers it to their care software. This step is necessary to add further information to the patient file, for example, information from patient conversations and decisions on care measurements.

This process can be time-consuming, as the care staff alternates between referring to the printout and typing the information into the system. During that time, confounding factors such as telephone ringing, colleagues, or technical issues can arise, prolonging the process.

Due to the use of different software in various facilities, the information is often displayed or organized differently, resulting in additional work.

Regarding the digital transmission of CTRs (cross-institutional dispatch and automatic integration into the in-house system), most (23/33, 70%) participants expressed no concerns. However, 30% (10/33) of them raised issues, such as concerns about possible threats to patient data protection (4/33, 12%). Most (24/33, 72%) respondents hope digital CTR transmission will reduce administrative effort. Some (18/33, 55%) participants indicated that they favored the standardization of CTRs because standardization of CTRs would result in relevant information being found more quickly in the future. On the basis of the responses, the primary consideration in developing a new solution should ensure, for example, that receiving, sending, and creating a CTR is less time-consuming for nurses than in the current process.

All (33/33, 100%) participants stated that CTR standardization would help them a lot as the CTRs they work with are usually different in structure and semantics.

Concerning the essential information in CTRs, all (33/33, 100%) participants agreed that patient information, medication, aids, and last bowel movement are considered to be very relevant regarding a potential standardization of CTRs. Finally, their opinion on automatic data integration was asked; they were curious as to whether something like this is possible so that they do not need to copy and paste information manually.

### Field Observations and Contextual Inquiries

#### Field Observations

The observations focused on the receiving side of the CTR, that is, the creation of a CTR in the in-house primary system. This means that the scenario of a receiving facility was always observed. This focus on the receiving facility was agreed upon through collaboration with the facilities due to the COVID-19 pandemic, as stricter visitor restrictions prevented parallel observation in both the sending and receiving facilities. In all cases, the transfer of a patient was announced in advance.

The observation occurred from the moment the nurse sat down at their computer to either create the patient case or fill it in. At the hospital, the cases are already created by the administration and contain information that is necessary for billing but does not influence the nursing documentation any further. One nurse was observed during every observation, but it was not necessarily every time the same as it depended on their schedule. While at the hospital, both field observations and contextual inquiries were conducted; only contextual inquiries took place in the care facility.

The results of field observations at University Hospital Augsburg (UHA; n=6) in 2020, showed a high administrative time burden for nurses (refer to Table 3 for the CTR transmission process). Manual recording of CTRs resulted in an average time expenditure of 34 minutes. The observations showed that the CTRs were not sent in advance but arrived with the patient. While entering the data into their in-house system, the care staff mentioned that they could not prepare adequately for the patient in advance (eg, by preparing medications and nursing aids). The field observation also showed that the nursing specialist endures many interruptions while entering the CTR (relatives, colleagues, physicians, telephone, patients, or emergency calls), forcing them to switch between different tasks very often. Therefore, the nurse had to refocus on the CTR repeatedly.

**Table 3:** Overview of care transition record (CTR) transmission process observations at the hospital.

Observation	Observation duration (min)	Software	Interruptions, n	Type of interruptions	Resource used for transferring data	CTR present (print)
1	50	ORBIS	5	Relatives, telephone, colleagues, physician, and missing information	Computer and telephone	Present
2	25	ORBIS	3	Telephone and missing information	Computer and telephone	Present
3	40	ORBIS	5	Relatives, telephone, colleagues, and ambulances	Computer and telephone	Present
4	45	ORBIS	4	Colleagues, physician, telephone, and patients	Computer	Present
5	25	ORBIS	1	Physician	Computer	Present
6	23	ORBIS	3	Emergency calls and colleagues	Computer	Present

There was no direct association between observation duration and the care need of a patient; rather, it depended on the overall setting, for example, completeness of the available information, number of interruptions, and the length of the CTR.

The CTRs encountered in the field observations were all from different nursing homes with different lengths (approximately 12 and 30 pages). The information is primarily unstructured, that is, free text. Structured elements were primarily areas with checkboxes.

During the observations, many of the observed nurses complained that the manual transfer of the CTR was time-consuming. After the observation, the care staff were asked additional questions regarding the relevance of a digital CTR process, the most important information to be transferred, and their opinion about the fully automatic integration of the CTR data into the in-house system. The questions were open ended and digitally documented by the observer. In terms of relevance, all participants (n=6) said that the early, preferably digital, transfer of the CTR would hold immense value. It would help them to prepare in advance and obtain, for example, missing information and medication beforehand. It would reduce their administrative workload. In total, 3 (50%) of the 6 participants stated that the current process is frustrating as patients are often transferred before the weekend without medication, physician’s notes, or aids. Without these things, they have to come up with makeshift solutions to care for the patients over the weekend.

After conducting the field observations at the hospital, it became evident that specific questions remained unanswered and could not be answered fully in the follow-up discussion. These questions were about the specific functionalities of the software used and also specific work-arounds that were conducted by the care staff but not remembered after the observation. Therefore, one additional contextual inquiry was conducted.

#### Contextual Inquiries

Contextual inquiries (n=5) were conducted in 2021-2022 at 2 care facilities and UHA (Table 4). The results of the contextual inquiries provided valuable insights into the observation duration, the confounding factors, and the aids used. For most observations, documents about the patient (eg, physician’s letter, medication plan, and CTR) were available as printouts. These were either sent with the patient or faxed to the referred institution. The latter could occur during registration or after inquiries about missing CTRs or information. The duration of 4 complete observations in care facilities and 1 hospital (excluding observation 2 because no input happened) averaged 47 minutes. In observation 2, it took 33 minutes to determine that no CTR was present, and it could not be sent from the sending facility. However, this required the nurse to make internal and external phone calls. She also needed to delegate procurement tasks to colleagues in the facility (eg, ask colleagues to check if the CTR might not be in the facility after all). In other cases, the CTR was handed out to the patient upon discharge but was not necessarily available right after the patient arrived at the receiving facility when the data were entered into the system.

**Table 4:** Overview of care transmission record (CTR) transmission process contextual inquiries, care facilities, and hospital. Care facilities are divided into facility 1 (CF1) and facility 2 (CF2).

Observation	Facility	Observation duration (min)	Software	Number of interruptions	Type of interruptions	Resource used for transferring data	CTR present (print)
1	CF1	55	Connext Vivendi NG+PD	1	Telephone, colleagues, and technical problems	Computer, smartphone, telephone, pen, and fax	Present
2	CF2	33	None used	0	No CTR present	Computer, smartphone, telephone, and fax	Not present
3	CF1	78	Connext Vivendi NG+PD	5	Telephone and colleagues	Computer, smartphone, telephone, paper, and pen	Present
4	Hospital	20	ORBIS	2	None	Laptop, paper, and patient	Present
5	CF2	37	Sic Pflege-assistent	3	Telephone	Telephone, paper, and pen	Present

All nurses involved in the contextual inquiries noted that the transfer process was time-consuming, particularly if they needed to retrieve missing information and also because they had to refocus on CTR data input due to interruptions.

Another interesting observation was that the nurses at the care facilities combined information from the CTR, physician’s letter, medication plans, and the initial interview with the patient and entered these in free-text fields.

After the contextual inquiries, the same questions were asked as in the field observations. The nurses responded very similarly.

Comparing the average observation duration of all care facilities (excluding observation 2 because no input happened) with the hospital shows that the care staff requires approximately 56 minutes in the care facilities and only 20 minutes at the hospital.

### BPMN Modeling

#### Overview

On the basis of the findings of the observations, BPMN models were created to better understand the various CTR activities (creating, sending, and receiving). These were discussed with the respective facility and detailed in the previous publication. After discussion, it was determined that the process models of the 2 care facilities can be combined into 1 process, as the activities are identical. Furthermore, the models were divided into different lanes, making it easier to understand which activities are manual and which are software based (human-computer interaction).

#### Transfer Process From Care Facility to Hospital

**Figure 1:**
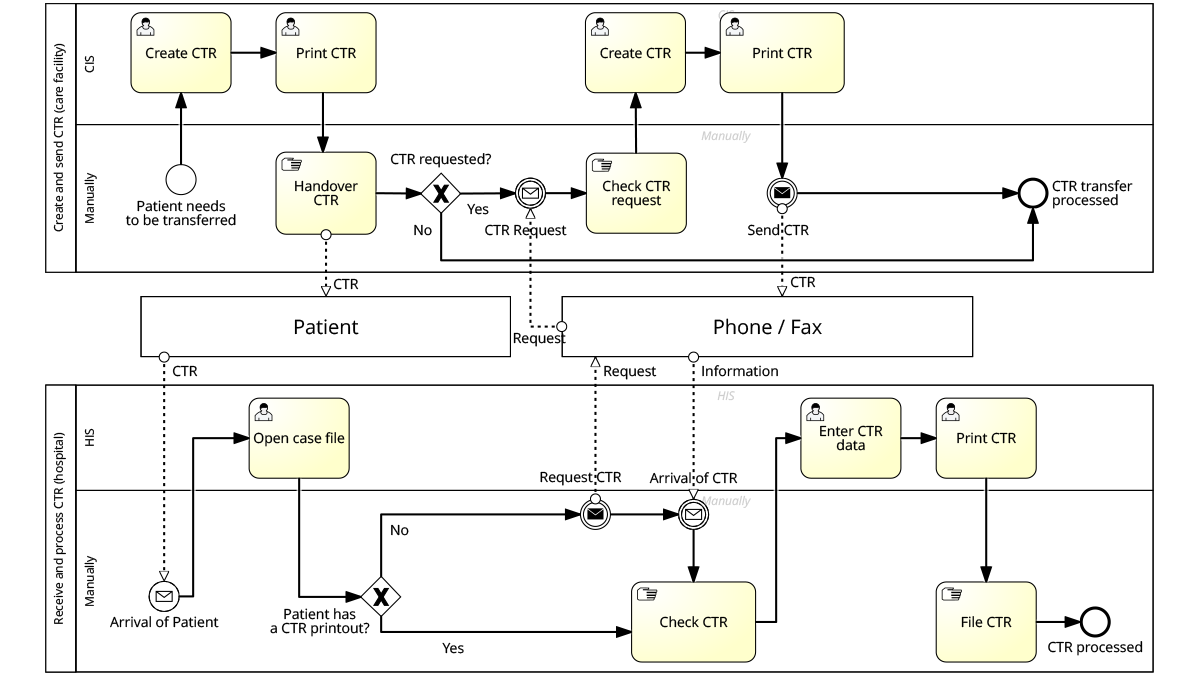
Process modeling: care transition record (CTR) data transfer from the care facility to the hospital. CIS: case information system; HIS: hospital information system.

The process starts with the patient’s need to be transferred (see Figure 1, Create and Send [Care Facility]). The nurse at the care facility creates a CTR, prints it, and usually hands it to the patient. Then, the patient arrives at the hospital, and the nurse at the hospital opens the patients’ case file (see Figure 1, Receive and Process CTR Hospital). Afterward, she checks if the CTR printout is available and whether it is complete and error free (referring to the content of the CTR). Patients’ case file is a digital file that contains the basic information of the patient for billing. As these files are prepared by the administration upon arrival of the patient, the care staff do not need to prepare those themselves.

If the CTR is complete and error free, she transfers the CTR into the hospital information system, prints the CTR in its specific structure, and files the CTR manually. After this, the CTR is processed, and the process is complete. If the CTR is unavailable, the nurse calls the sending care facility. The request is then processed there. If a CTR is missing, the sending facility creates a CTR, prints it, and sends it via fax to the hospital. Next, the nurse checks the document (eg, the correct CTR for the patient). After that, the CTR is transferred into the hospital information system, printed, and manually filed. Then, the process is complete.

#### Transfer Process From Hospital to Care Facility

**Figure 2:**
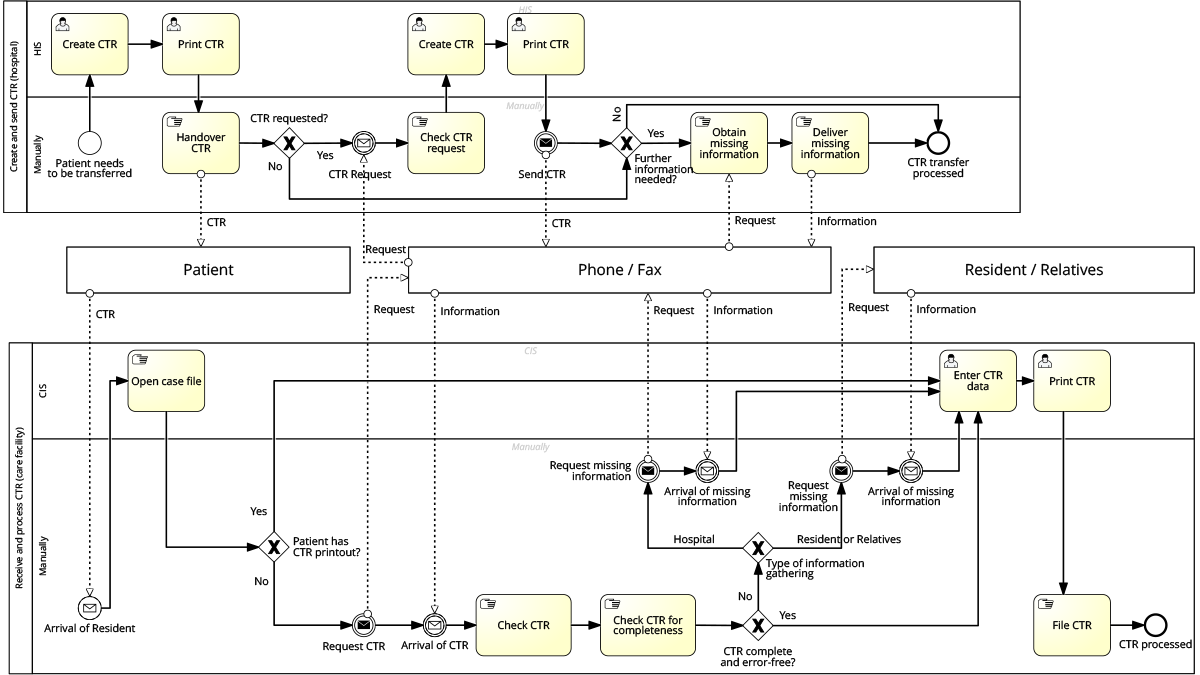
Process modeling: care transition record (CTR) data transfer from hospital to care facility. CIS: case information system; HIS: hospital information system.

A patient is transferred from the UHA (Figure 2) to a care facility. At the UHA, the nurse creates a CTR, prints it, and hands it to the patient. Upon arrival of the patient (now called resident), the nurse at the care facility logs into their care information system and checks if the resident has a printed CTR. If so, they start transferring the CTR into the system. Afterward, they print the document in a proprietary file format and file it manually, then the process is complete.

If the CTR is unavailable, the nurse requests it from the UHA via phone. In the UHA, the request is checked and processed. A CTR is created, printed, and transferred via fax. Upon receipt of the missing CTR, the nurse checks whether it is the correct CTR for the resident and verifies its completeness and validity. If there is missing or incorrect information, the nurse requests the missing information either from the UHA via telephone or directly through the resident or relatives (this option does not exist in the process at the hospital). After the arrival of the missing information, the nurse starts transferring the CTR, prints it from the care information system, and manually files it. The CTR is processed, and the process is complete.

### Semantic Analysis of CTRs

**Figure 3:**
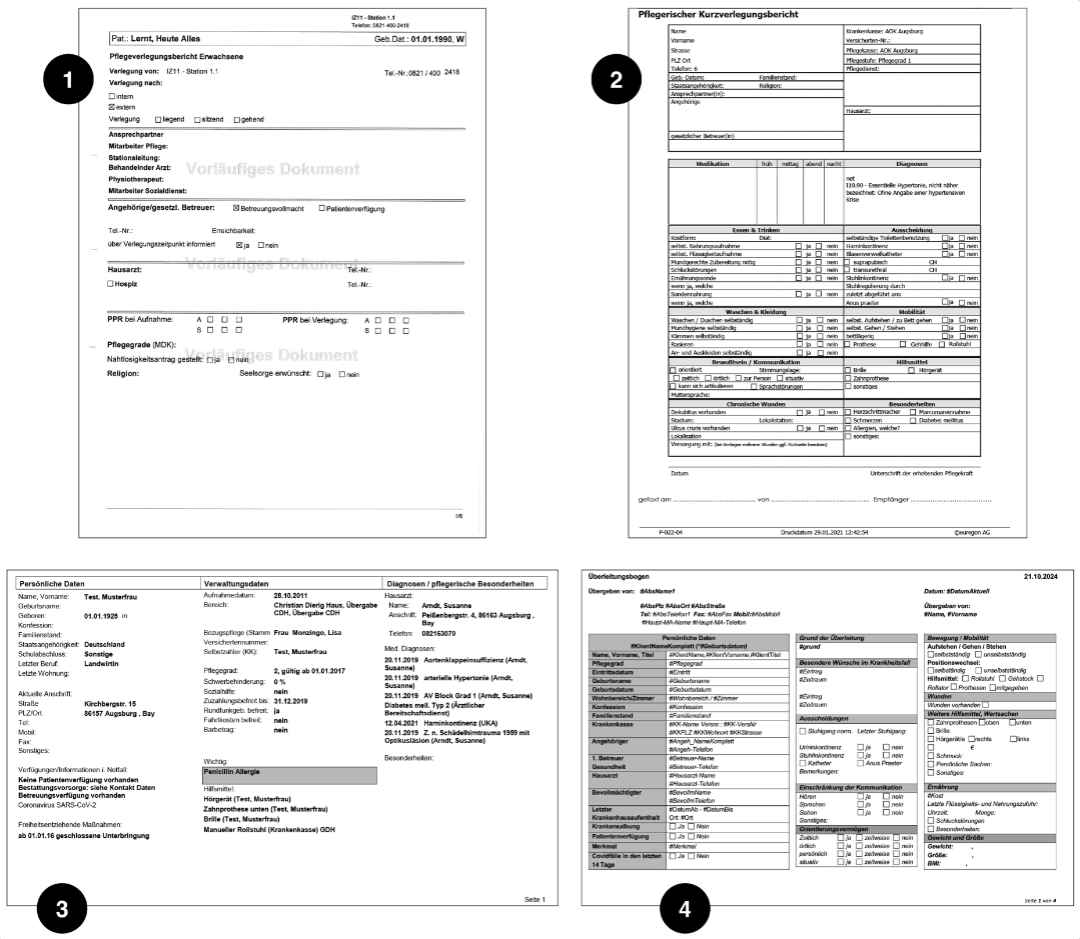
First page of care transition records (CTRs) from one hospital (1) and 3 care facilities (2-4).

A total of 4 CTRs of cooperation facilities were analyzed regarding their structures, similarities, and differences. The analysis highlighted their different structures (eg, bowel movement on the front page or second or third page) or different wordings (eg, movement or mobility). The analysis and follow-up meetings with care staff revealed that this makes it challenging to work with CTRs effectively, as some of the most important fields are located at the end of the report. The meetings also revealed that the CTRs from the hospital are typically shorter (≤8 pages) and hold more structured information (checkboxes) than free-text fields. In comparison, CTRs from the care facilities are usually longer (≤20 pages) and include more free-text fields. The front pages of each analyzed CTR are shown in Figure 3, showcasing their different structure.

**Figure 4:**
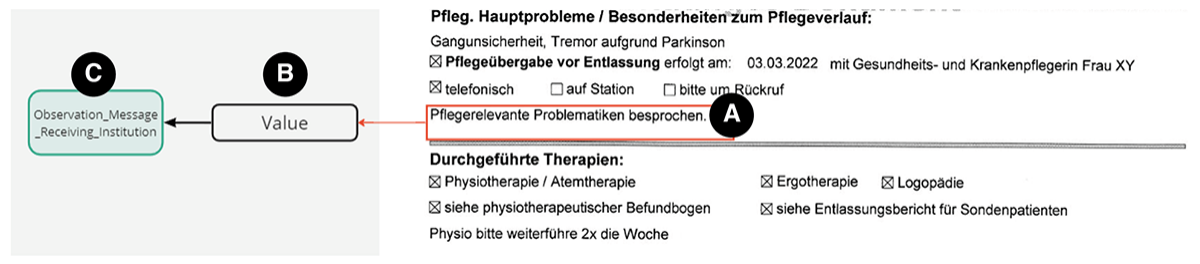
PDF care transition record (CTR) on the right with a free-text field (A, red rectangle) mapped to corresponding resources on the left (B, black and green rectangle, C).

In the next step, a semantic analysis, including the mapping of CTRs to the new CIO-CTR standard, was conducted. This was done by assigning parts of the CTR to the data structure of the CIO-CTR (Figure 4). The green box represents a CIO-CTR resource, the white box represents the specification of the resource, and the red box represents the information of the CTR.

Throughout the process, it was realized that the mapping often cannot be done straightforwardly. There were some entities (eg, diagnosed diseases, deafness, aphasia, and limited vision) that could not be assigned to a single field in the CIO-CTR. This was mainly because some of the resources of the CIO-CTR format were too similar to each other. Most of the issues with overlapping assignability were resolved by further study of the CIO-CTR standard and discussion with the research team. For uncertain cases, meetings with mio42 GmbH (originator of the format) were held. Decisions regarding mappings were then based on their feedback. Nevertheless, in some cases, an assignment was still not possible. There was no resource element that provided information about whether the patient or resident had been transferred within a facility (internal; transmission, eg, within a hospital from one to another department) or outside (external; transmission from another facility).

Furthermore, some fields in the CIO-CTR are implemented as free-text fields, which makes unambiguous, error-free mapping difficult.

**Figure 5:**
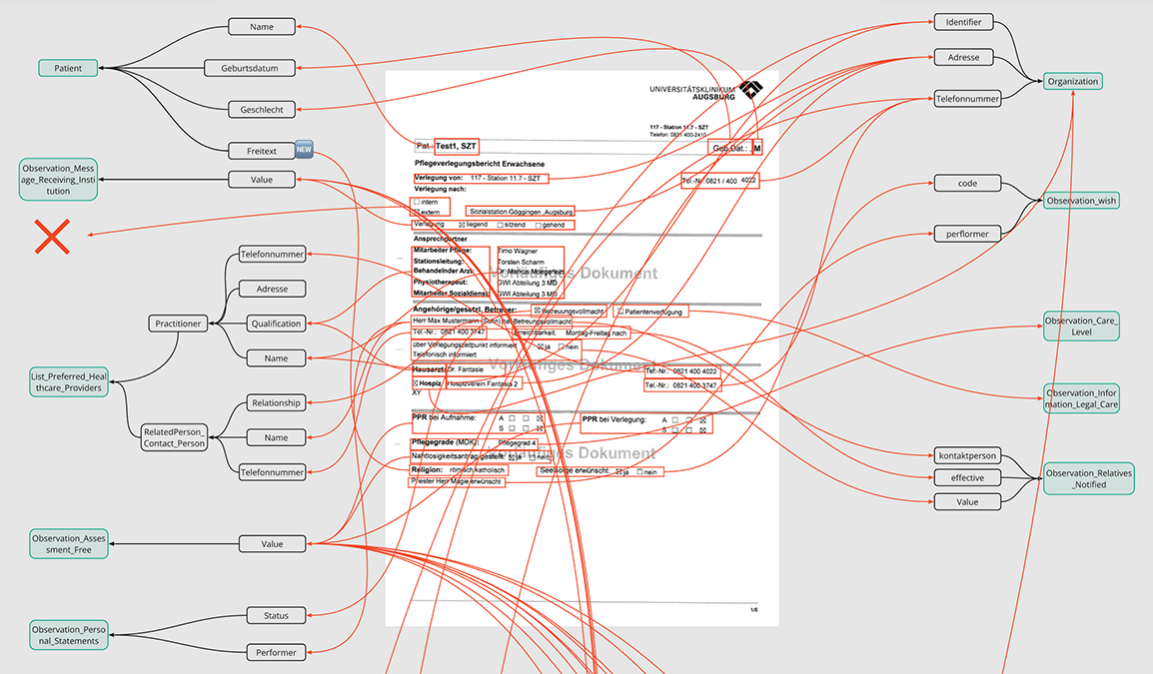
Mapping 1: excerpt of the mapping of a care transition record (CTR) of the University Hospital Augsburg to the care information object (CIO) CTR standard (1 of the 6 pages). The X shows that one piece of information could not be mapped.

**Figure 6:**
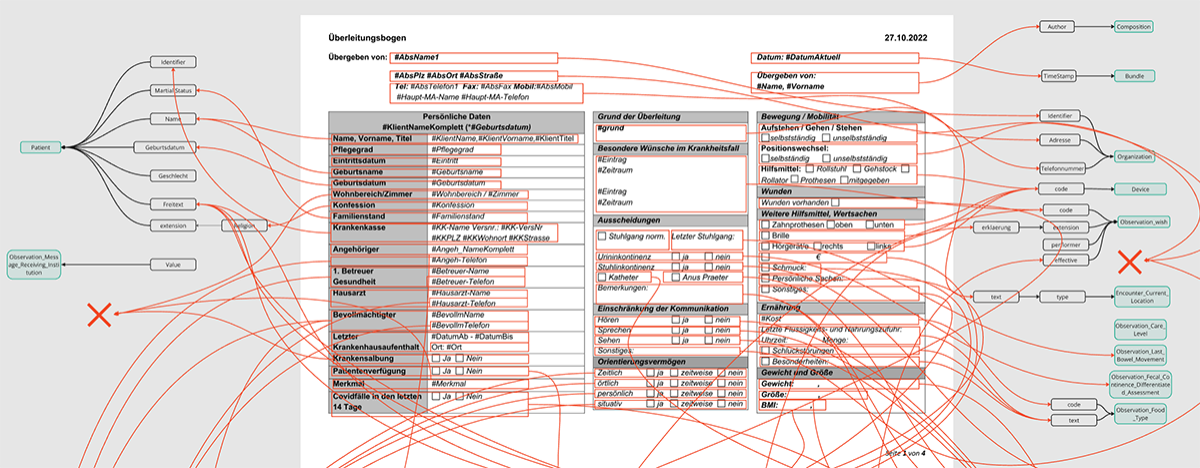
Mapping 2: excerpt of the mapping of a care transition record (CTR) of a care facility to the Pflegerisches Informationsobjekt (PIO) standard (1 of the 4 pages). The X shows that two pieces of information could not be mapped.

An excerpt of the mapping of site-specific CTRs of 2 facilities to the new standard CIO-CTR is shown subsequently. Mapping 1 focuses on the CTR of the UHA (Figure 5), and mapping 2 focuses on the CTR of 1 care facility (Figure 6). The visuals illustrate the overall complexity and difficulty of mapping each entity correctly. In mapping 1, it was possible to assign 147 (99.3%) of the 148 information objects from CTR to the CIO-CTR; in mapping 2, it was only possible to map 114 (91.2%) of the 125 information objects.

This raises the question of what should be done with the information that could not be mapped. One possibility would be to add it to the free-text fields.

### Frequency Analysis of CTR Entities at UHA

The occurrence of individual data entities in 204 CTRs of UHA and 54 CTRs from care facilities was analyzed to find out their frequencies. As comparable field entries are needed for processing, only the CTRs of UHA were used for subsequent processing, as this dataset was the biggest.

An entity is understood as a single piece of information represented in the CTR by its input field.

On the basis of these results, a percentage for each entity was computed (entity is filled or not filled), and a frequency range was created (commonly used, occasionally used, and rarely used). These ranges estimate the frequency of entities in the nursing transition process and are shown as follows: (1) 100% to 50%: commonly used entities, (2) 49% to 25%: occasionally used entities, and (3) 24% to 0%: rarely used entities

The results of each entity were presented to care staff (n=2) at UHA who are involved in the CTR process for discussion. An extract of the results is presented in Table 5. It is important to note that the frequency analysis was limited to data that did not include personal information about patients (eg, date of birth, primary care physician, contact options, and religious affiliation), as the UHA anonymized the CTRs before further processing. However, during the discussion, the nursing staff stated that all personal data could be classified as very relevant.

**Table 5:** Extract from the frequency analysis from University Hospital Augsburg care transition records (N=204).

				Frequency, n (%)
**Very relevant (100%-50%)**				
	Ability of self-body care		201 (98.5)	
	Orientation ability		198 (97.1)	
	Dressing		197 (96.6)	
	Medication: reference to physician’s letter		195 (95.6)	
	State of consciousness		190 (93.1)	
	Nutrition		188 (92.2)	
	Mobility		179 (87.7)	
	Presence of pain		177 (86.8)	
	Main diagnosis		167 (81.9)	
	Last bowel movement		149 (73.0)	
	Items brought along (suitcase)		121 (59.3)	
	The degree of care		115 (56.4)	
**Relevant (49%-25%)**				
	Nursing-relevant secondary diagnoses		69 (33.8)	
	The location of the pain		68 (33.3)	
	The special features of the care process		58 (28.4)	
**Less to not relevant (24%-0%)**				
	Medication: reference to a medication plan		23 (11.3)	
	Free-text field about pain		19 (9.3)	
	Seamless request (yes or no)		17 (8.3)	
	Pastoral care requested (yes or no)		3 (1.5)	
	Aids ordered and their retailers		1 (0.5)	
	**Items brought along**			
		Valuables	23 (11.3)	
		Insurance card	22 (10.8)	
		Identification	5 (2.5)	
		Patient passport	0 (0.0)	

Although the information about the main diagnosis (167/204, 82.3%), state of consciousness (190/204, 93%), and nutrition (188/204, 92%) occurs with high frequency in the dataset, their placement in the paper-based CTR is inadequate, as they appear relatively late in the document.

Another finding is that bowel movement is rated as an essential piece of information (149/204, 73%), but 55/204 (27%) do not include it in the CTR.

Medication information was also expected to be present more frequently; however, because this information is usually included in the physician’s letter rather than in the CTR, the occurrence was only 11% (23/204).

Regarding items brought along, many selection possibilities were given in the UHA’s CTR. Valuables (23/204, 11%) and insurance cards (23/204, 11%) had the highest frequency among them. However, no additional information about the individual items could be provided.

### User Story Mapping

There were 2 workshops conducted, involving a total of 7 participants. These participants were part of the core research project team, bringing diverse expertise from various disciplines: health care (n=2, 29%), computer science (n=3, 43%), design (n=1, 14%), and IT security (n=1, 14%). All 7 (100%) participants attended both workshops, ensuring continuity and consistency in the discussions and decisions. During the first workshop (hybrid, due to COVID-19 restrictions), participants used both physical materials (paper and whiteboards) and digital tools (Zoom [Zoom Communications] and chat) to record potential user stories. The process involved writing down ideas and then engaging in a collaborative card-sorting exercise to discuss and prioritize these stories. A whiteboard was used to document the structured user journey, which was shared with online participants via camera, ensuring everyone had equal access to the visual information.

Between the first and second workshops, participants had approximately 2 weeks to reflect on the identified user stories and their potential impact on the development process. This period allowed the team to refine their understanding and prepare for the next stage of discussion, which focused on a stepwise implementation plan (release planning).

**Figure 7:**
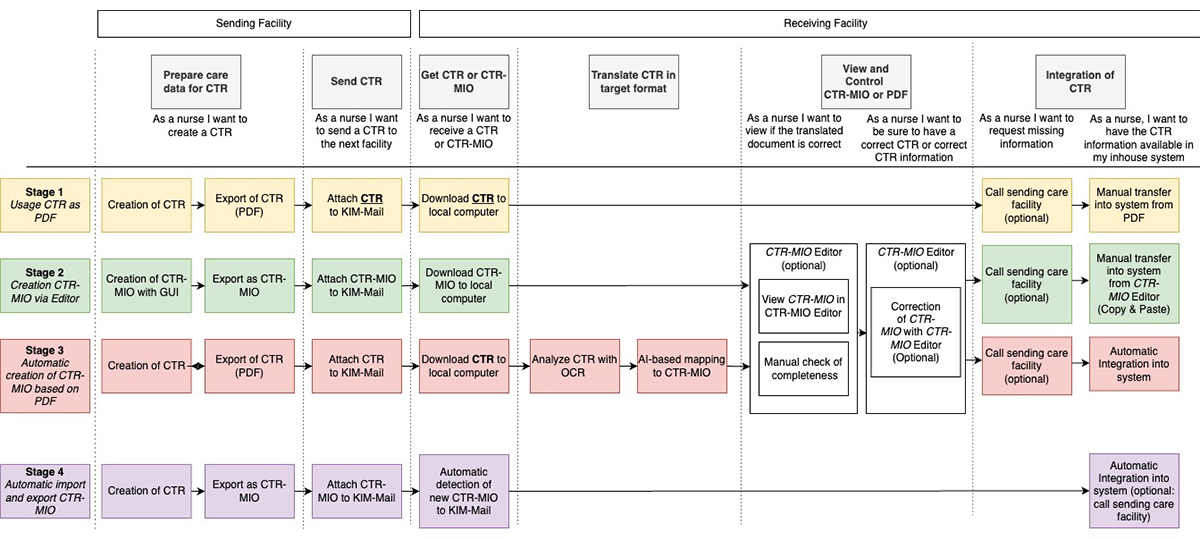
Final user story mapping with 4 release stages (on the left). Colors are used to better distinguish between the stages. Some story cards do not have color, as they apply to multiple stages. AI: artificial intelligence; CIO: care information object; CTR: care transition record; KIM: Kommunikation im Medizinwesen.

The second workshop was conducted entirely online using the tools Zoom and Miro. Miro is an online collaborative platform developed by RealtimeBoard Inc. The participants had time to share their reflections on the previous work, discuss it, and refine their understanding of the user journey. Afterward, the participants focused on creating release stages to guide the upcoming development. They collaboratively designed the user story map due to the workshops, which was continuously refined throughout the project. The final result, a road map proposal, can be seen in Figure 7. The Backbone section describes the backbone and the proposed solution’s release stages.

In Figure 7, four release stages are shown on the left and divided into 4 colors to provide a better division throughout the user stories. Some story cards do not have color, as they apply to multiple release stages. The subsequent sections describe the use dimensions, backbone, release stages, and implementation scenarios.

### Backbone

The horizontal axis of the user story map shows the main activities that have to be performed sequentially to achieve care data exchange between facilities. These activities are referred to as epics and are listed in the top row of Figure 7. The user stories concretize the epics. One activity (translating CTR in target format) has to be performed only in an intermediate release stage, and it becomes obsolete as soon as all facilities use the standardized target format.

### Release Stages

A brief overview of all 4 release stages can be seen in Figure 7. A description of each stage is given in the subsequent sections.

#### Release Stage 1: Use of CTR in PDF Format

The first release requires the least implementation effort but already meets one basic requirement: timely, digital transfer via the TI. The functionality is limited to conventional CTRs, typically in PDF format. This release requires both the sending and receiving care facilities to be connected to the TI. As usual, the sending facility creates a CTR in its facility-specific layout and transmits it using the KIM service. The receiving facility can then retrieve the CTR from its KIM mailbox.

#### Release Stage 2: Creation of CIO-CTR via Editor

At the beginning of a transition process to the new standardized CIO-CTR format, few or no in-house systems will support the new standard. To remain independent from software manufacturers, a dedicated software module that can create, read, and edit CTRs in the new format (“CIO-CTR editor”) would be beneficial. Nurses could use this editor to create CIO-CTRs and send them to the receiving institution via KIM. Particular emphasis should be placed on the user-centered design of the interface, particularly regarding the structure of the input options and how information is compiled. This could serve as a blueprint for later implementation in the proprietary software systems.

#### Release Stage 3: Automatic Creation of CIO-CTR Based on PDF

Another, more complex way to create and transfer a CTR in the new standard format is to transform the conventional, proprietary CTR using an automatic artificial intelligence (AI)–based tool. On the basis of the previous analysis of CTRs, it can be assumed that most CTR data will be unstructured and provided in PDF format. A transformer service could analyze this structure using AI and extract text sections with an optical character recognition module. The extracted content is then mapped to the CIO-CTR format. This approach would be relevant if a receiving facility is already capable of processing CIO-CTRs but receives a nonstandardized CTR via KIM. With a transformer, the new CTR-CIO format can be generated and imported with little extra effort.

#### Release Stage 4: Automatic Export and Import of CIO-CTR

In this final stage, the care staff can create a CTR in the in-house system, export it as a CIO-CTR, and transfer it via the TI. After receiving the CIO-CTR, the receiving facility can then integrate it directly into their in-house system. The benefit is that neither a transformer service nor a separate editor would be needed, resulting in the least effort for the care staff. This requires the software manufacturers of the various care and medical information systems to fully support the new CIO-CTR format; however, it is unclear when this will happen.

## Discussion

### Principal Findings

Despite years of efforts toward digitalization in health care in Germany, our research shows that the creation and transmission of CTRs remain highly time-consuming, averaging 34.67 (SD 10.78) minutes at hospitals and 44.6 (SD 20.5) minutes in care facilities (findings from observations).

#### Semantic Interoperability of CTRs Between Institutions

As health care systems transition toward digital formats, it becomes increasingly important to enable different institutions to exchange, understand, and use the transmitted data seamlessly. From the perspective of nursing science, discharge management has long been recognized as a crucial aspect of patient care. Efficient discharge processes ensure that patients receive continued care, reduce readmission rates, and improve overall patient outcomes. The CIO-CTR standard, introduced in December 2022, marks a significant step toward a fully digital exchange of CTR data. However, our study reveals that this progress has been hampered by a lack of widespread implementation and resistance. Because the CIO-CTR is not legally binding and the necessary updates are resource intensive for software manufacturers, they prefer to concentrate on more urgent issues. Thus, we propose an iterative, stepwise implementation approach that could gradually improve the situation.

#### Iterative Implementation Approach

The user story map with the resulting release stages offers a step-by-step approach toward a seamless digital solution. As the overall issue is complex, changes cannot be expected simultaneously at all ends. A quick, early solution is the mere digital transfer of CTRs in existing, proprietary formats via a digital infrastructure (stage 1). For this, the institutions only have to be connected to the health data network (TI), as they are obliged by law in Germany by July 1, 2025 (according to §341 (8) SGB V [46]) and a KIM account is set up. Sending CTRs in the institutions’ traditional formats does not require them to have updated software that can read or export the new CIO-CTR format. At this stage, the time-consuming manual data transfer into the in-house systems is still required. The goal is for all software systems in all institutions to directly import and export CTRs in the new format, and for all the information to be integrated automatically into in-house systems (stage 4). During a transition time, when only some of the systems can process CTRs in the new format, certain incompatibilities will occur, which we want to address with interim solutions: the CIO-CTR viewer or editor (stage 2) and the CTR-transformer (stage 3).

For stage 1 (data transfer via TI), we accompany and assist our cooperating partner institutions in installing the necessary infrastructure to connect to the TI. In this regard, we plan to offer experience reports, which could lower the entry hurdle, particularly for care facilities.

For stage 2, we are developing an open-source software where CIO-CTRs can be created, viewed, and edited. This has several benefits: (1) developing an editor with a concrete suggestion for a user interface visualizing the CIO-CTR standard provides a figurative basis for discussion between developers, care professionals, and regulatory institutions; (2) bridging the gap for continuous digital transfer if not all institutions support the new digital standard; and (3) serving as a blueprint for software manufacturers who want to implement the new CIO-CTR.

Stage 3 introduces an automated process to convert CTRs from proprietary formats (eg, scanned PDFs) into the CIO-CTR format, using AI-based mapping. This solution is applicable when an institution that can process CIO-CTRs but receives a nonstandardized CTR. Of course, this automatic transfer would have to be reliable, and creating such a component would be complex, as many different proprietary formats exist, and as seen in the semantic mapping, a direct transfer is not possible in all cases.

Stage 4 represents the most desirable solution. Nurses would be able to work with an improved process without manual transfer of CTR data, potentially leading to a minimization of disruptions. The primary responsibility for implementing the CIO-CTR falls on system manufacturers. To facilitate this transition, the manufacturers could actively be supported by providing a reference implementation for the new standard, for example, conducting workshops and organizing related events. This collaborative effort would support a smooth and efficient integration of the CIO-CTR into existing systems while minimizing the burden on health care providers.

#### Contributions

Our research used an iterative, user-centered methodological approach to develop a road map that helps overcome the current challenges in the CTR transmission process in Germany. This road map offers a practical, phased approach toward digital solutions, particularly valuable in settings where full-scale adoption of digital standards is not yet feasible. It provides health care providers with a flexible pathway to transition toward digital care processes without requiring immediate, costly system changes. This road map is more than the mere definition of a new format; it supports gradual digital integration.

#### Future Implications and Work

If the adoption of digital standards remains voluntary and lacks regulatory support, the duration required to establish a standard data format is likely to be prolonged. Without more vigorous regulatory enforcement and widespread buy-in from all stakeholders, the vision of seamless care transitions may remain out of reach. Therefore, future efforts must focus not only on technological solutions but also on fostering collaboration between regulators, software providers, and health care institutions to ensure the long-term success of health care digitalization.

Our current solution uses KIM as a means of transport within the TI. As soon as the ePA is more widely adopted in Germany, an exchange of the CTR via this means might be preferable over KIM. Future work has to investigate this further.

### Limitations

The COVID-19 pandemic posed significant challenges for data collection, particularly in gaining access to cooperative facilities. The necessary planning and multiple postponements due to visitor restrictions limited our ability to observe the complete care transition process.

The pandemic may have also introduced a selection bias in our web-based questionnaire. Nurses facing higher technical barriers or those under significant stress due to pandemic-related demands may have been less likely to participate, which could skew the findings toward participants who were more technically adept or had fewer pandemic-related pressures.

Furthermore, the sample size of the web-based questionnaire (n=33 usable datasets), while offering valuable qualitative insights, limits the generalizability of our findings.

Our study used a qualitative research approach to gain in-depth, context-specific insights. The combination of field observations, contextual inquiries, and questionnaire data from hospital and care facilities provided a rich understanding of the practical barriers and opportunities in transitioning to digital CTR processes. The relatively small sample sizes for field observations (n=6) and contextual inquiries (n=5) were sufficient for this research’s detailed, exploratory nature but could limit the robustness of the conclusions.

### Conclusions

A future solution should simplify the overall CTR transmission process by minimizing the manual transfers into the in-house systems, standardizing the CTR, and providing a secure digital transfer. Doing so could positively impact the overall care process and patient experience. With our suggestion for a stepwise solution, we attempt to make the complex task feasible, ultimately supporting care staff with their daily activities and processes.

## Abbreviations

AI: artificial intelligence
BPMN: business process model and notation
CIO: care information object
CTR: care transition record
ePA: electronic patient record
GDPR: General Data Protection Regulation
HL7: Health Level 7
KIM: Kommunikation im Medizinwesen
TI: telematics infrastructure
UHA: University Hospital Augsburg
